# Study of Toxic Metals and Microelements in Honey as a Tool to Support Beekeeping Production and Consumer Safety

**DOI:** 10.3390/foods14111986

**Published:** 2025-06-04

**Authors:** Clara Naccari, Vincenzo Ferrantelli, Gaetano Cammilleri, Giuseppe Barbaccia, Pietro Riolo, Maria Carmela Ferrante, Antonio Procopio, Ernesto Palma

**Affiliations:** 1 Department of Health Sciences, University “Magna Græcia” of Catanzaro, 88100 Catanzaro, Italy; procopio@unicz.it (A.P.); palma@unicz.it (E.P.); 2 Istituto Zooprofilattico Sperimentale della Sicilia “A. Mirri”, 90129 Palermo, Italy; vincenzo.ferrantelli@izssicilia.it (V.F.); gaetano.cammilleri86@gmail.com (G.C.); giuseppe.barbaccia@izssicilia.it (G.B.); pietro.riolo@izssicilia.it (P.R.); 3 Department of Veterinary Medicine and Animal Productions, University of Federico II, 80137 Napoli, Italy; ferrante@unina.it; 4 AGreen Food Research Center, University “Magna Græcia” of Catanzaro, 88100 Catanzaro, Italy; 5 Interdepartmental Service Center for Pharmacological Research, Food Safety, High Tech and Health (CIS-IRC-FSH), University “Magna Græcia” of Catanzaro, 88100 Catanzaro, Italy

**Keywords:** honey, toxic metals, microelements, MRLs, risk assessment parameters, RDA, ICP-MS, EU regulations, apiculture

## Abstract

Background: Honey is a beekeeping product with high nutritional value, considered a bio-indicator of environmental pollution. The aim of this study was to determine the mineral content in honey by analyzing toxic metals in accordance with EU regulations and evaluating the intake of microelements through honey consumption. Methods: Honey samples of different floral origins were subjected to ICP-MS analysis for the determination of toxic metals and metalloids (Cd, Pb, As) as well as microelements (Cu, Zn, Se, Fe, Mn, Co, and Al). The data were considered significant for *p*-values < 0.05. Results: All analyzed minerals were detected above the limit of detection (LOD) in every sample. Among toxic metals, lead (Pb) levels exceeded the maximum residue limit (MRL) of 0.1 mg/kg, as established by EU Regulation 2023/915, in most samples. However, these levels corresponded to a small percentage of the Provisional Tolerable Weekly and Daily Intake. The concentrations of microelements significantly contributed to the Recommended Daily Allowance (RDA). Conclusions: This study documents the presence of toxic metals in the analyzed honey, with lead (Pb) levels exceeding the MRL. The microelement content provides adequate nutritional intake through honey consumption. Therefore, studying the mineral profile can be used to monitor environmental pollution in the areas where the apiaries are located and to assess the safety of honey.

## 1. Introduction

Honey is the primary beekeeping product, traditionally consumed for its high nutritional value [1,2], as it is rich in sugars, proteins, amino acids, vitamins, minerals, phenolic acids, flavonoids, organic acids, and aromatic substances [3,4].

Due to its complex composition, honey is considered a nutraceutical food with several beneficial properties for human health, such as antioxidant, anti-inflammatory, and antimicrobial [5,6], as well as laxative, re-mineralizing, energizing, emollient, and immune-stimulating activities [7]. It is also used to treat sore throats and as an adjuvant in conditions such as asthenia, anorexia, weight loss, intestinal infections, anemia, and intoxications [8,9,10].

Honey is considered a sustainable food, naturally produced by honeybees (*Apis mellifera*) with minimal environmental impact. It is derived from flower nectar [11] or from secretions and excretions of plant parts, which the bees collect and transform by combining with specific substances of their own. They then deposit, dehydrate, store, and leave the mixture in the honeycomb to ripen and mature [12]. The final honey is a fresh product, obtained without processing, additives, or modifications by beekeepers [13]. Its composition is directly correlated with the floral origin of the nectar, as well as environmental and climatic conditions, which contribute to qualitative differences among honey varieties.

Honey is also considered a useful bio-indicator of environmental pollution, used to assess the presence of inorganic contaminants (e.g., heavy metals) and organic pollutants (e.g., pesticides, dioxins) [14,15,16,17,18,19]. Honey and other beekeeping products are often exposed to these contaminants depending on the environmental pollution where the apiaries are located, primarily due to industrial activities and agricultural practices near the beehives where bees forage for nectar [20,21].

Among the pollutants, metals are present in the environment due to natural processes (e.g., soil erosion, volcanic eruptions) and human activities (e.g., agriculture, industrial operations, waste disposal). These metals can diffuse from the soil into plants via their root systems [22], and subsequently be transferred by bees into honey and other apicultural products, where they remain as residues.

Since bees can travel up to 5–10 km during foraging flights, they are highly susceptible to environmental contaminants from polluted plants, soil, water, and air [23,24]. As a consequence of the diffusion in the environment, metals can easily enter the food chain with serious risks to human health. Most in detail, metals are the natural minor constituents of honey but, particularly, Fe, Zn, Se, Mn are essential microelements, responsible for some benefic effects for health, being involved in detoxifying processes and immune defenses [25], although they can become potentially toxic at high concentrations.

Heavy metals, toxic elements also at low concentrations, can influence honeybee behavior and reproduction and are easily spread by bees in apicultural products. Particularly, the presence of toxic metals in honey can alter its mineral profile and, consequently, affect the quality of this apicultural product. Therefore, this aspect is an object of global interest to support beekeeping farms, considering that honeybees are pollinators for 80% of flowering plants [11] and producers of a very required food in the human diet.

For the important rule of honey as a nutraceutical in diet, especially of children, ill and old persons, particular attention is focused on both toxicological and nutritional aspects, to quantify the metals exposure through honey consumption and the balance among toxic metals and essential microelements [26,27].

The aim of this study was to determine the mineral content in honey samples of different floral origin, to analyze the residual levels of toxic metals according to EU Regulations and specific risk assessment parameters, and to evaluate the microelements intake through honey assumption, to support the quality of beekeeping production and guarantee the consumer’s safety.

## 2. Materials and Methods

### 2.1. Reagents, Chemicals and Gases

All solutions were prepared using ultra-pure analytical-grade reagents. Ultra-pure deionized water (resistivity of 18.2 MΩ·cm) was obtained using a Milli-Q^®^ Integral water purification system with Q-Pod (Millipore, Bedford, MA, USA); Ultra-pure nitric acid (60% *v*/*v*) was purchased from Merck KgaA (Darmstadt, Germany). Standard solutions of each element (1000 mg/L), of ICP-MS grade and traceable to NIST, were purchased from VWR International LTD (Randon, PA, USA), and from each single element were prepared multi-elements calibration solutions at different concentration levels (range 0.05–10 µg/L). To optimize the analytical performance, a Tuning Solution for ICP-MS, capable of covering a wide range of masses (Ce, Co, Li, Mg, Tl, and Y 1 µg/L) was purchased from Agilent Technologies (Santa Monica, CA, USA). Internal Standard stock solutions (100 mg/L) of scandium (Sc), yttrium (Y), indium (In), terbium (Tb), rhodium (Rh), lutetium (Lu), lithium (Li), indium (In), germanium (Ge) and bismuth (Bi) were purchased from Agilent Technologies (Santa Monica, CA, USA). The carrier gas argon (Ar), the dilution gas helium (He), and hydrogen (H_2_), all of the ultra-pure grade (99.9995%), were purchased from SOL S.p.a. (Monza, Mi, Italy).

### 2.2. Sampling and Sample Preparation

The analysis was carried out on honey samples of different floral origins, Wildflower (n = 10), Citrus (n = 8), Chestnut (n = 8), Honeydew (n = 6), Erika (n = 6), collected from several beekeeping farms in Calabria region (Italy). Samples were freshly collected during the active beekeeping season (April to September) from apiaries located in rural areas of the Calabria region, specifically in the provinces of Catanzaro and Vibo Valentia.

All samples were stored in clean, sealed glass jars and kept in the dark at 4 °C until to analysis. All samples were submitted to acid digestion according to the method of Naccari et al., 2015 [28] (in accordance with the UNI EN 13805:2002). Aliquots of 0.5 g of honey were weighed and transferred into PTFE-TFM vessels (polytetrafluoroethylene), previously washed with 60% ultra-pure nitric acid (*v*/*v*) and ultra-pure water and digested with HNO_3_ (5 mL) and H_2_O_2_ (1 mL) using a Multiwave 3000 microwave digestion system (Anton Paar, Graz, Austria). Analytical blanks were prepared in the same manner, without honey samples, to check for potential contamination during analysis. Subsequently, the digested honey samples were diluted to 50 mL with ultra-pure water and filtered through 0.45 µm syringe filters to minimize interference.

### 2.3. ICP-MS Analysis

The analysis of toxic metals and metalloids (Cd, Pb, As) and microelements (Cr, Ni, Co, Mn, Fe, Cu, Zn, Se, and Al) was carried out using an ICP-MS (7700x series, Agilent Technologies, Santa Monica, CA, USA), equipped with octopole reaction system (ORS3), with a peristaltic pump for sample solution introduction from tubes arranged on an auto-sampler ASX-500 Series (Agilent Technologies, Santa Monica, CA, USA) and combined with a quartz cyclonic chamber (water-cooled, 2 °C), according to the method of Messina et al., 2025 [29]. The specific instrumental conditions of the ICP-MS analysis performed were reported in Table 1. Each sample was analyzed three times, in the presence of a blank to correct the background signals. Analyses were conducted by constructing a calibration curve (range: 0.05–10 µg/L) using seven standard concentrations, with linear interpolation, a maximum allowable error of 5% per standard, and a correlation coefficient exceeding 0.99.

### 2.4. Validation Method

The analytical method was validated according to the parameters described in ISO 17025:2018. To calculate the instrumental limits of detection and quantification (LOD and LOQ), the 3σ and 10σ approaches were used. The reliability of the method was evaluated through the recovery of three concentration levels (0.005–0.05–0.5 mg/kg) and accepted with a range between 90 and 110% for each analysis.

### 2.5. Statistical Analysis

The statistical analyses were computed in Microsoft Excel and GraphPadPRISM (version 9.0, GraphPad program Inc., La Jolla, CA, USA). The Principal Components Analysis (PCA) has been carried out to evaluate the contribution of each element to the total variance of samples and to differentiate honey samples using R 4.4.2 software.

Data are expressed as mean values ± standard deviation (S.D.) from at least three replicates. Differences were considered statistically significant at *p* < 0.05.

### 2.6. Parameters of Toxicological Risk Assessment and Nutritional Evaluation

For the toxicological assessment of exposure to inorganic contaminants through honey consumption, the residual levels of toxic metals and metalloids found in samples analyzed were expressed as a percentage of Provisional Tolerable Weekly Intake (PTWI), Provisional Tolerable Monthly Intake (PTMI) and Provisional Maximum Tolerable Daily Intake (PMTDI), established by JECFA, calculated for an adult of 70 kg b.w. and considering the average per capita honey consumption currently reported at 700 g per year [30], equivalent to approximately 1.92 g per day.

For each element, the Estimated Daily Intake (EDI) was also calculated using the following formula:

EDI = (C × IR/BW)(1)

where C is the concentration of each metal detected in honey samples (mg/kg), IR is the daily intake of honey (1.92 g/day), and BW body mass (70 kg for an adult).

The Target Hazard Quotient (THQ), which expresses the non-carcinogenic risk of long-term exposure to individual metals, was calculated using the following formula:

THQ = EDI/R*f*D*m* (2)

where the R*f*D*m* is the oral reference dose (mg/Kg b.w./day). The R*f*D*m* used were 0.0035 for Pb, 1 for Al, 0.0003 for As, 0.0001 for Cd, 0.003 for Cr, 0.10 for Mn, 0.3 for Zn, 0.7 for Fe, as proposed by Flamminii et al. 2024 [31], considering the variations in thresholds of several minerals.

Other parameters considered for both nutritional and toxicological evaluations were the Dietary Reference Values (DRVs), established by the European Food Safety Authority (EFSA, 2019) [32], for copper (Cu), iron (Fe), manganese (Mn), selenium (Se), and zinc (Zn), calculated for an adult of 70 kg b.w. and the assumption of 1.92 g/day of honey (ISMEA 2024) [30]. More specifically, to evaluate the potential nutritional contributions and toxicological implications of the essential trace metals detected in the honey samples, a comparison was carried out between the mean concentrations of selected elements (mg/kg) and their DRVs. As defined in the EFSA Summary Report on DRVs for nutrients [32], DRVs are considered an umbrella term encompassing parameters such as Adequate Intake (AI), Average Requirement (AR), Population Reference Intake (PRI), Reference Intake Range (RI), and Tolerable Upper Intake Level (UL). The EDI of Cu, Fe, Mn, Se, and Zn were calculated by multiplying the respective mean concentrations found by the average honey consumption of 1.92 g/day. The resulting intake values were compared with EFSA thresholds for different population groups (including infants, children, adults, and pregnant and lactating women), to determine whether daily intake from honey contributes significantly to nutrient requirements or approaches potentially harmful levels. Risk characterization was based on the relative proportion of intake compared to both AI/PRI and UL for each element. Finally, for nutritional evaluation of honey’s contribution to mineral intake through the diet, the concentrations of essential microelements found in Calabrian samples were expressed as a percentage of Recommended Daily Allowance (RDA), fixed in Europe for an adult (30 µg/day for Cr; 55 mg/day for Se; 2.3 mg/day for Mn; 8 mg/day for Fe; 900 µg/day for Cu; 11 mg/day for Zn; 45 µg/day for Mo) [33] and actually revisited by the National Library of Medicine of USA.

## 3. Results

### 3.1. Toxic Metals and Metalloids and Microelements in Honey Samples

The results, reported in Table 2 and Table 3, indicated the presence of all analyzed toxic metals, metalloids, and microelements above the limit of detection (LOD) in the honey samples. The ICP-MS method demonstrated an acceptable linear regression model for the trace elements analyzed, with a strong correlation coefficient (r^2^ = 0.999). The LOD and LOQ values ranged from 0.001 to 0.005 mg/kg, complying with Regulation (EC) No. 333/2007 on sampling and analysis methods for official control of heavy metals in food. Recovery rates ranged from 96% to 105%.

Among the toxic metals (Table 2), the highest residual levels were found for Pb (range: 0.0792–0.1250 mg/kg), lowest for As (range: 0.0026–0.0128 mg/kg) and Cd (range: 0.00632–0.0090 mg/kg). The residual levels of toxic elements were similar in all samples and, however, higher in Wildflower and Citrus and lowest in Chestnut honey samples.

The concentrations of microelements are reported in Table 3. Aluminum (Al) was the most abundant element, followed by zinc (Zn), manganese (Mn), iron (Fe), and copper (Cu), while selenium (Se), cobalt (Co), nickel (Ni), and chromium (Cr) were detected at lower concentrations.

**Figure 1:**
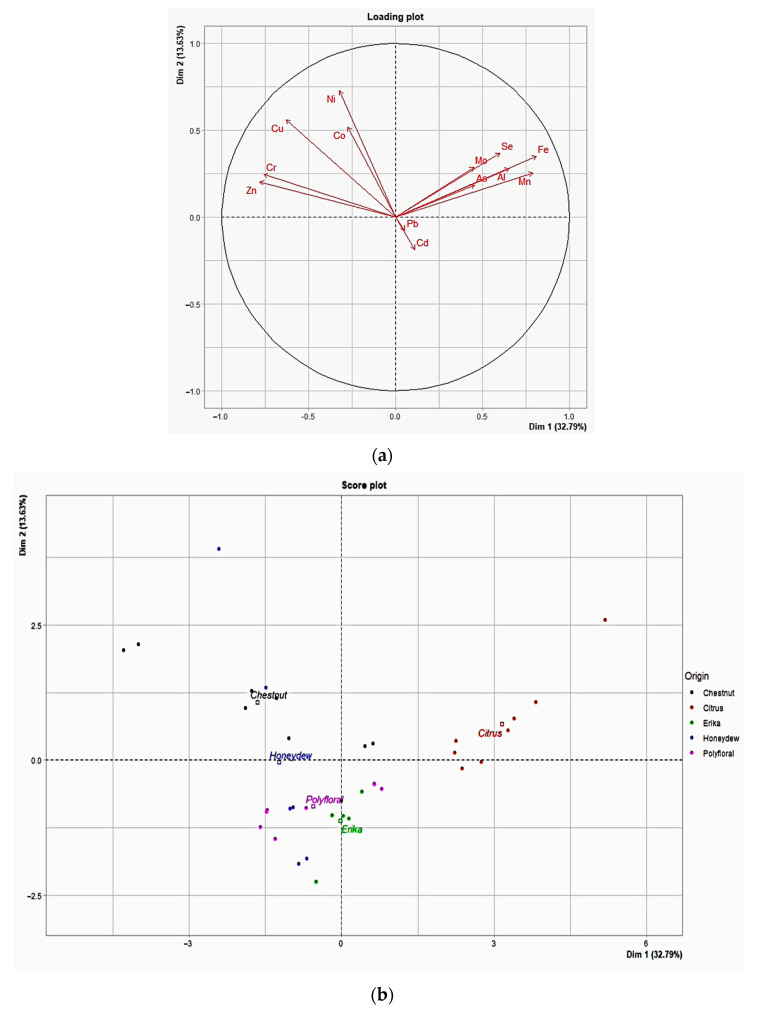
PCA score plot (**a**) and loading plot (**b**) of the trace metals and metalloids loadings (vectors) and the individual scores of PC1 and PC2 according to the honey types examined.

Principal Component Analysis (PCA) was performed, and the PC1 vs. PC2 score plot and loading plot are shown in Figure 1a,b, respectively. The first principal component (PC1) explained 32.79% of the variance, whereas the second (PC2) was 13.63%, indicating 46.42% of the total variance. PCA revealed that Citrus honey samples were clearly distinguishable from the others, indicating unique chemical characteristics. Chestnut and Honeydew kinds of honey appeared more overlapping, suggesting similarities between these two types of honey. Erika and Wildflower kinds of honey formed distinct clusters. Fe, Al, Mn, Pb, and Cd appeared to have a significant weight along PC1, indicating that PC1 represents a combination of these variables. Regarding PC2, Ni, Co, and Cu were notable, as they contributed significantly along this axis. Pb and Cd showed a strong positive correlation, reflecting similar concentration trends in the samples. Conversely, Zn and Ni have opposite angles compared to some of the main variables, suggesting a negative correlation with them. Fe and Al were located close to each other with similar orientations, indicating that their variation was closely correlated. The Citrus kinds of honey were distinct along PC1, suggesting that their composition in Fe, Mn, Pb, and Cd was unique compared to the others. The Chestnut and Honeydew samples showed an overlap along PC1 and PC2, suggesting that these honeys may have similar chemical compositions or significant overlaps.

### 3.2. Toxicological Assessments and Nutritional Assessment

**Figure 2:**
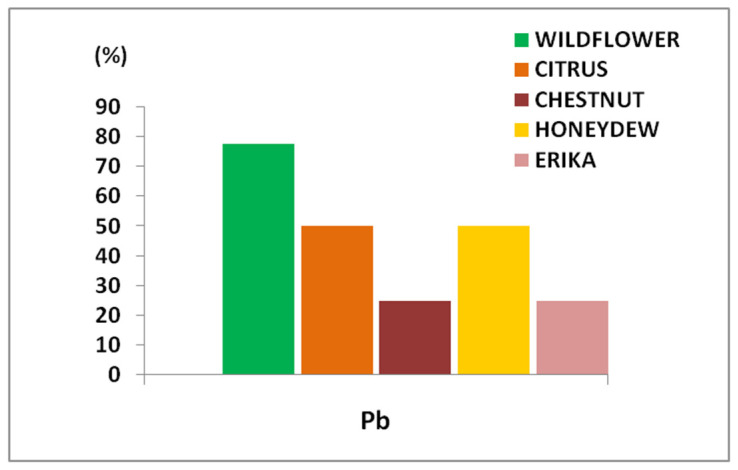
Percentage of honey samples exceeding the specific MRL (0.1 mg/kg) fixed for Pb in apicultural products.

For a more detailed toxicological assessment, the most significant data concerned lead (Pb). In most of the analyzed samples, Pb levels exceeded the maximum residue limits (MRL) of 0.1 mg/kg, as established by EU Regulation 2023/915 for honey and apicultural products (Figure 2).

**Figure 3:**
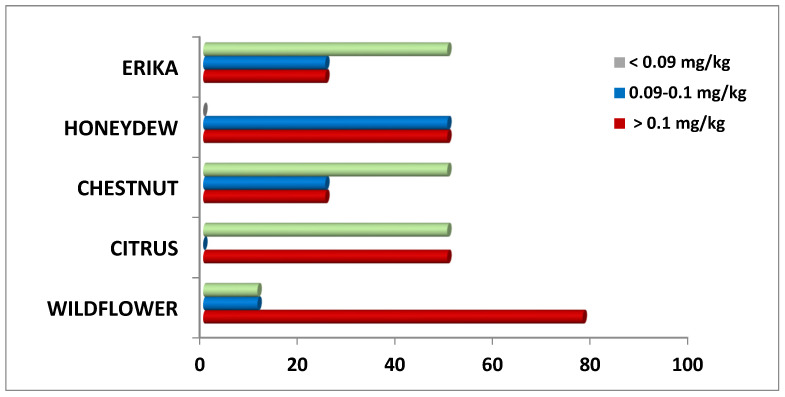
Distribution (%) of Pb content in honey samples of different floral origin.

In more detail, considering the distribution of Pb content in these honey samples, it is possible to observe some important differences (Figure 3). Specifically, 80% of Wildflower samples exceeded the MRL, followed by 50% of Citrus and Honeydew samples, and 25% of Erika and Chestnut samples. Therefore, Wildflower honey resulted in the most contamination while Erika and Chestnut the least.

For food safety, the specific parameters of risk assessment were taken into consideration, particularly PTWI, PTMI, and PMTDI. These parameters were proposed by the Joint FAO/WHO Expert Committee on Food Additives (JECFA)—World Health Organization (WHO) for heavy metals and metalloids, toxic elements at low concentrations, and essential microelements, potentially toxic only in higher concentrations (Table 4). These parameters are submitted periodically for evaluation by these agencies, in consideration of new sensitive and accurate methods available, able to confirm or propose new safe and protective values for human health, as reported in Table 4.

The concentration of all minerals analyzed in Calabrian honey samples was expressed as a percentage of specific parameters of risk assessment, PTWI, PTDI, and PTMI; in addition, also the EDI and THQ were calculated. As shown in Table 5, the residual levels in honey samples represented a small percentage of these reference parameters.

In Table 6, instead, the Dietary Reference Values (DRV) and all parameters correlated are reported according to EFSA indications, relating to essential trace elements present in Calabrian honey samples, considering the honey average consumption in Italy. From these results, it is possible to observe that none of the estimated intake approach or exceed the recommended or tolerable thresholds across population groups for metals considered Cu, Fe, Mn, Se, and Zn.

Finally, for the important role of honey as a nutraceutical in the diet, the concentrations of essential microelements found in samples analyzed were expressed as a percentage of Recommended Daily Allowance (RDA), fixed in Europe for an adult of 70 kg [33]. As reported in Table 7, the concentration of essential microelements found in all honey samples contributed to the mineral daily intake. For this calculation, it was considered an estimated consumption of 1.92 g/day/person of honey (ISMEA 2024), corresponding to a moderate assumption of honey due to the high content of sugars. These data show that consumption of the analyzed Calabrian honey samples contributes to the dietary intake of essential minerals, particularly chromium (Cr) and copper (Cu).

## 4. Discussion

The study of mineral content in honey is object of interest for the possible toxicological risk of exposure to toxic metals and metalloids and for the health and nutritional aspects, attributable to the presence of microelements. As honey is a widely consumed apicultural product, monitoring studies are useful to evaluate variations in toxic metal content as indicators of environmental pollution near beekeeping farms and to verify the complete mineral profile for assessing the product’s quality and safety.

In the mineral content of honey, it is possible to observe differences according to the honey varieties, plant species that bees forage, geo-morphological characteristics of soil where plant species grow, environmental conditions of areas where apiaries are located, etc. [24,34].

This study confirmed the presence of toxic metals and metalloids in the analyzed honey samples. Among all elements studied, the highest levels of Pb found could be correlated to the environmental pollution of areas where beekeeping farms are sited. The Calabrian farms were located in rural areas employed for intensive agriculture but possibly subject to the influence of anthropogenic activities and vehicular traffic from the nearby roads [35,36]. Particularly Pb, being a low-mobility metal, is present mainly in polluted air [37], therefore, Pb presence in honey is attributable to bee exposure during flights in search of food.

Given that bees travel long distances, they may be exposed to inorganic contaminants through plants, soil, water, and air [23], subsequently transferring these elements into the honey. Instead, the microelements are needed for plant growth and are adsorbed through the radical apparatus from the soil [38]; particularly, among all elements considered, the content of Se and Al is influenced by the soil composition and specific geographical areas.

From a comparative analysis with data present in the literature, the mineral profile of the kinds of honey from Calabria analyzed in this study was lower than samples from Colorado [39], Spain [40], Morocco [41], Turkey [3,42], Hungary [43], United Kingdom [44], Saudi Arabia [45] but similar to samples from the United States [46] and North-western Italy [13].

Regarding toxic metals, the most noteworthy finding was that Pb levels exceeded the MRL established by EU regulations for honey and apicultural products in the majority of the analyzed samples [47]. Particularly, the highest percentages of Pb, due to environmental pollution, were found in Wildflower honey, probably in relation to the floral origin. In fact, the honey variety depends on the nectar of the different flowers and on the interactions among geographical features, climate, and environmental conditions of the production areas [13,48]. As a multifloral product, Wildflower honey presents a characteristic mineral profile and properties influenced by the dominant pollen species [49]. It may also be more exposed to inorganic contaminants found in plant materials compared to mono-floral varieties, as documented by other authors [13].

The toxicological risk assessment was carried out to evaluate dietary exposure to these inorganic contaminants through honey and the potential health risks to consumers. The content of metals and metalloids found in the honey samples were correlated to specific parameters, PTWI, PTDI, and PTMI, proposed by the Joint FAO/WHO Expert Committee on Food Additives (JECFA)—World Health Organization (WHO) for each metal, in consideration of possible risk of exposure. The residual levels of all metals and metalloids found in the honey samples of this study corresponded to a low percentage of these parameters, demonstrating that the consumption of Calabrian kinds of honey does not contribute significantly to metals exposure with the diet. In addition, the calculation of EDI and THQ showed small values not significant for metals risk assessment. In fact, according to EPA guidelines US EPA, 2002 [50], THQ ≥ 1 is indicative of adverse health effects, therefore, values of THQs below 1 found for all metals analyzed in this study were not dangerous for human health.

Furthermore, a comparison among EDI, calculated for each essential trace metal in the contest of Italian dietary exposure using updated per capita consumption data, and EFSA’s DRVs across relevant population groups, was carried out to better evaluate the potential nutritional contribution and toxicological implications of these elements. Data obtained showed that the estimated intakes resulting from the average honey consumption were consistently well below EFSA reference values, confirming that honey does not represent a significant risk of excessive exposure to these metals.

Considering more in detail the nutritional aspect, honey samples analyzed resulted rich in microelements and the concentrations found, expressed as percentages of RDA, fixed in Europe for an adult of 70 kg and actually revisited by the National Library of Medicine of USA, demonstrated that the assumption of this food gave a significant contribution to intake of Cr and Cu, according to the floral origin [51,52]. In addition, the abundance of essential microelements in honey, particularly Se, Zn, Cu, and Fe, for their antioxidant and detoxifying properties, contributes to increasing the immune response and also to counteract metal toxicity in humans [53].

The results of this study confirmed that honey, being a trade of union between animal and plant kingdoms, is a valid bio-indicator of environmental pollution, according to *One Health* approach. As documented by statistical analysis of the distribution of these metals, it was possible to observe that toxic metals contributed to a lesser extent to the total variance of samples, being environmental pollutants. Instead, the PCA differentiated the honey samples on the basis of the floral origin, and, specifically, Citrus samples were characterized by a high content of Se, Fe, and Mn, while Chestnut samples mainly by Ni, Co, Cu, Mn, and Zn.

In accordance with other authors [54,55], our data showed that, among essential metals, Mn plays a key role in the identification of honey varieties. The Chestnut honeys analyzed in this study were clearly distinguishable for Mn content, together with Ni, Co, Cu, and Zn; instead, in a study carried out on samples from northern Italy the high influence of botanical origin was attributable to Mn, Al, Cu, and Rb [13], present in highest concentrations. Other authors found very high Mn levels in Slovenian chestnut honey, considering this metal as the specific marker for botanical species rather than for geographical origin [56]. However, Mn levels of this study resulted intermediate compared to literature data, lower than in Chestnut honey from Australia [23] but higher than those reported by Solayman et al., 2016 [25]. The Citrus kinds of honey, instead, were clearly differentiated for the high content of Mn, Fe, and Se, according to the availability of metals from soil and our data resulted in intermediate compared to other authors [57,58].

Relating to Al, a non-essential metal and toxic to biological systems with serious negative effects for both humans and animals, the concentrations found in the analyzed honey were the highest among all elements but lower than data reported in the literature [3,41]. This data is mainly correlated to environmental pollution, which is one of the most abundant elements present in the soil. In addition, data from the literature reported that Al content in honey could be influenced also by the aluminum equipment used during the processing of honey before marketing [3].

## 5. Conclusions

This study documented the presence of toxic metals and metalloids in all honey samples and the presence of Pb residual levels higher than MRL, possibly due to pollution of areas where apiaries were located, posing potential risks to consumers, although the concentrations of these metals corresponded to a low percentage of specific parameters of risk assessment. The content of essential microelements found in the same honey samples, instead, resulted in a normal range, compared to literature data, and adequate to contribute to minerals nutritional intake through honey consumption. Therefore, the study of the mineral profile can be considered valid to monitor the environmental pollution of areas near apiaries and to assess the quality and safety of honey for consumers’ health.

## Figures and Tables

**Table 1:** Instrumental conditions of the ICP-MS analysis.

Parameter	Value
RF-Power (W)	1500
Carrier gas flow (mL/min)	1.2
Plasma gas flow (mL/min)	15
Auxiliary gas flow (mL/min)	1.0
Spray chamber	Water-cooled double pass
Spray chamber temperature (°C)	2
Lens voltage	4.5
Mass resolution	0.7
Integration time points/ms	3
Points per peak	3
Replicates	4

**Table 2:** Toxic metals and metalloids (mg/kg w.w.) in honey samples of different floral varieties.

Honey Samples		Pb	Cd	As
Wildflower (n = 10)	Mean	0.125	0.008	0.003
	S.D.	0.085	0.003	0.002
	Median	0.934	0.006	0.002
Citrus (n = 8)	Mean	0.124	0.009	0.013
	S.D.	0.066	0.005	0.001
	Median	0.111	0.008	0.009
Chestnut (n = 8)	Mean	0.079	0.006	0.003
	S.D.	0.043	0.002	0.011
	Median	0.075	0.005	0.003
Honeydew (n = 6)	Mean	0.109	0.008	0.006
	S.D.	0.013	0.002	0.001
	Median	0.103	0.007	0.004
Erika (n = 6)	Mean	0.104	0.007	0.007
	S.D.	0.004	0.001	0.001
	Median	0.091	0.006	0.006

**Table 3:** Levels of microelements (mg/kg w.w.) in honey samples of different floral varieties.

Honey Samples		Se	Zn	Cu	Fe	Mn	Co	Ni	Cr	Al
Wildflower (n = 10)	Mean	0.024	2.114	0.372	1.058	1.210	0.016	0.071	0.036	4.466
	S.D.	0.009	0.102	0.109	0.548	0.097	0.004	0.017	0.006	0.498
	Median	0.013	1.983	0.457	1.750	1.052	0.018	0.076	0.035	4.172
Citrus (n = 8)	Mean	0.562	1.288	0.351	2.755	5.716	0.012	0.079	0.011	6.037
	S.D.	0.036	0.078	0.022	0.265	0.559	0.005	0.006	0.002	0.915
	Median	0.178	1.311	0.358	2.776	5.695	0.012	0.078	0.018	6.133
Chestnut (n = 8)	Mean	0.050	2.313	1.910	1.056	2.896	0.019	0.108	0.086	4.764
	S.D.	0.015	0.210	0.283	0.039	0.042	0.003	0.012	0.024	0.814
	Median	0.059	2.352	1.937	1.069	2.948	0.020	0.109	0.087	4.626
Honeydew (n = 6)	Mean	0.212	1.905	0.878	1.623	3.274	0.016	0.086	0.044	5.089
	S.D.	0.005	0.047	0.081	0.081	0.065	0.009	0.023	0.008	0.372
	Median	0.014	1.722	0.688	0.830	0.547	0.013	0.187	0.032	4.982
Erika (n = 6)	Mean	0.275	1.836	1.046	1.811	3.962	0.016	0.091	0.047	5.297
	S.D.	0.007	0.047	0.010	0.169	0.013	0.001	0.005	0.001	0.287
	Median	0.018	1.469	0.233	1.354	0.158	0.003	0.043	0.036	5.031

**Table 4:** Parameters of risk assessment proposed by Joint FAO/WHO Expert Committee on Food Additives (JECFA) (http://apps.who.int/food-additives-contaminats-jecfa-database/Home/Chemical accessed on 11 November 2024).

PTWI (Provisional Tolerable Weekly Intake)				
Pb	PTWI 0.025 mg/kg	JECFA 1993	PTWI withdrawn. Not possible to establish a new PTWI protective for health	Evaluation 2011
Cd	PTWI 0.007 mg/kg bw	JECFA 2003	PTMI 25 µg/kg b.w./month	Evaluation 2010, confirmed 2021
As	PTWI 0.015 mg/kg bw	JECFA 1988	PTWI 15 µg/kg b.w. withdrawn	Evaluation 2011
Al	PTWI 1 mg/kg bw	JECFA 1988	PTWI 2 mg/kg b.w.	Evaluation 2011
**PMTDI (Provisional Maximum Tolerable Daily Intake)**				
Se	PMTDI 9.4 μg/kg bw/day	JECFA 2011	PTWI 66 μg/kgb.w./week	JECFA-WHO, 72 (2011)
Zn	PMTDI 0.3-1 mg/kg bw/day	JECFA 1992	PTWI 7mg/kg b.w./week	JECFA 1992
Cu	PMTDI 0.5 mg/kg bw/day	JECFA 1992	PTWI 3.5mg/kg bw/week	JECFA 1992
Fe	PMTDI 0.8 mg/kg bw/day	JECFA 1983	-	**-**
Mn	PMTDI 0.36mg/kg bw/day	JECFA 1992	PTWI 2.5 mg/kg b.w./week	JECFA 1992

**Table 5:** Residual levels of toxic and essential metals expressed as PTWI (%), PTDI (%), PTMI (%), PMTDI (%), EDI (mg/Kg/day), and THQ for 1.92 g/day of honey (ISMEA 2024).

Element	Parameter	Wildflower	Citrus	Chestnut	Honeydew	Erika
Pb	PTWI	1.40 × 10^−2^	1.30 × 10^−2^	9.00 × 10^−3^	1.20 × 10^−2^	1.10 × 10^−2^
	EDI	3.00 × 10^−6^	2.00 × 10^−4^	3.00 × 10^−6^	2.00 × 10^−4^	3.00 × 10^−6^
	THQ	9.70 × 10^−4^	9.60 × 10^−4^	6.10 × 10^−4^	8.50 × 10^−4^	8.10 × 10^−4^
Cd	PTDI	2.97 × 10^−5^	3.26 × 10^−5^	2.28 × 10^−5^	2.82 × 10^−5^	2.79 × 10^−5^
	EDI	2.00 × 10^−7^	2.00 × 10^−7^	2.00 × 10^−7^	2.00 × 10^−7^	1.00 × 10^−7^
	THQ	2.23 × 10^−3^	2.44 × 10^−3^	1.71 × 10^−3^	2.12 × 10^−3^	2.09 × 10^−3^
As	PTWI	5.00 × 10^−4^	2.30 × 10^−3^	6.00 × 10^−4^	1.10 × 10^−3^	1.40 × 10^−3^
	EDI	1.00 × 10^−7^	3.00 × 10^−7^	1.00 × 10^−7^	2.00 × 10^−7^	2.00 × 10^−7^
	THQ	2.40 × 10^−4^	1.16 × 10^−3^	3.00 × 10^−4^	5.60 × 10^−4^	6.80 × 10^−4^
Al	PTDI	9.00 × 10^−4^	1.20 × 10^−3^	9.00 × 10^−4^	1.00 × 10^−3^	1.00 × 10^−3^
	EDI	1.20 × 10^−4^	1.60 × 10^−4^	1.30 × 10^−4^	1.40 × 10^−4^	1.40 × 10^−4^
	THQ	1.20 × 10^−4^	1.60 × 10^−4^	1.30 × 10^−4^	1.40 × 10^−4^	1.40 × 10^−4^
Se	PTMI	2.60 × 10^−3^	6.10 × 10^−2^	5.40 × 10^−3^	2.30 × 10^−2^	2.98 × 10^−2^
	EDI	1.00 × 10^−6^	1.50 × 10^−5^	1.00 × 10^−6^	6.00 × 10^−6^	7.00 × 10^−6^
	THQ	-	-	-	-	-
Zn	PTMI	2.30 × 10^−1^	1.40 × 10^−1^	2.51 × 10^−1^	2.07 × 10^−1^	1.99 × 10^−1^
	EDI	5.70 × 10^−5^	3.50 × 10^−5^	6.30 × 10^−5^	5.20 × 10^−5^	5.00 × 10^−5^
	THQ	1.90 × 10^−4^	1.20 × 10^−4^	2.10 × 10^−4^	1.70 × 10^−4^	1.70 × 10^−4^
Cu	PMTDI	3.00 × 10^−4^	3.00 × 10^−4^	1.50 × 10^−3^	7.00 × 10^−4^	8.00 × 10^−4^
	EDI	1.00 × 10^−5^	1.00 × 10^−5^	5.20 × 10^−5^	2.40 × 10^−5^	2.80 × 10^−5^
	THQ	2.50 × 10^−4^	2.40 × 10^−4^	1.30 × 10^−3^	6.00 × 10^−4^	7.10 × 10^−4^
Fe	PTMI	1.15 × 10^−1^	2.99 × 10^−1^	1.15 × 10^−1^	1.76 × 10^−1^	1.97 × 10^−1^
	EDI	2.90 × 10^−5^	7.50 × 10^−5^	2.90 × 10^−5^	4.40 × 10^−5^	4.90 × 10^−5^
	THQ	4.00 × 10^−5^	1.10 × 10^−4^	4.00 × 10^−5^	6.00 × 10^−5^	7.00 × 10^−5^
Mn	PTMI	1.30 × 10^−3^	6.20 × 10^−3^	3.10 × 10^−3^	3.60 × 10^−3^	4.30 × 10^−3^
	EDI	3.30 × 10^−5^	1.55 × 10^−4^	7.90 × 10^−5^	8.90 × 10^−5^	1.08 × 10^−4^
	THQ	3.30× 10^−5^	1.55 × 10^−4^	7.90 × 10^−5^	8.90 × 10^−5^	1.08 × 10^−4^

**Table 6:** Estimated daily intake of essential trace elements detected in honey of different floral varieties, expressed in mg or µg per day, considering the average consumption (1.92 g/day) compared to EFSA Dietary Reference Values (AI, AR, PRI, RI, UL).

Element	Honey Type	Conc. Found (mg/Kg)	EDI from Honey (mg/day)	AI	AR	PRI	UL	Risk
Cu	Wildflower	0.372	7.10 × 10^−4^	0.4–1.6 mg/day	NA	NA	1–5 mg/day	None
	Citrus	0.351	6.70 × 10^−4^					None
	Chestnut	1.911	3.67 × 10^−3^					None
	Honeydew	0.878	1.69 × 10^−3^					None
	Erika	1.046	2.01 × 10^−3^					None
Fe	Wildflower	1.058	2.03 × 10^−3^	5–16 mg/day	5–12.7 mg/day	7–16.3 mg/day	ND	None
	Citrus	2.755	5.29 × 10^−3^					None
	Chestnut	1.056	2.03 × 10^−3^					None
	Honeydew	1.623	3.12 × 10^−3^					None
	Erika	1.811	3.47 × 10^−3^					None
Mn	Wildflower	1.210	2.32 × 10^−3^	0.5–3 mg/day	NA	NA	ND	None
	Citrus	5.716	1.10 × 10^−2^					None
	Chestnut	2.896	5.56 × 10^−3^					None
	Honeydew	3.274	6.28 × 10^−3^					None
	Erika	3.962	7.61 × 10^−3^					None
Se	Wildflower	0.024	4.61 × 10^−2^	15–85 µg/day	NA	NA	45–255 µg/day	None
	Citrus	0.174	3.34 × 10^−1^					None
	Chestnut	0.050	9.60 × 10^−2^					None
	Honeydew	0.083	1.59 × 10^−1^					None
	Erika	0.102	1.96 × 10^−1^					None
Zn	Wildflower	2.115	4.07 × 10^−3^	2.4–12.7 mg/day	2.4–12.7 mg/day	2.9–16.3 mg/day	7–25 mg/day	None
	Citrus	1.289	2.47 × 10^−3^					None
	Chestnut	2.313	4.44 × 10^−3^					None
	Honeydew	1.905	3.66 × 10^−3^					None
	Erika	1.836	3.52 × 10^−3^					None

NA = not applicable; ND = not determined. Estimated daily intake (EDI); Adequate Intake (AI); Average Requirement (AR); Population Reference Intake (PRI); and Tolerable Upper Intake Level (UL).

**Table 7:** Levels of essential microelements expressed as a percentage of Recommended Daily Allowance (RDA) (https://www.ncbi.nlm.nih.gov accessed on 11 November 2024) for 1.92 g/day of honey (ISMEA 2024).

Honey Samples	Wildflower	Citrus	Chestnut	Honeydew	Erika
Cr % RDA 30 µg/day	0.227	0.069	0.547	0.206	0.232
Cu % RDA 1 mg/day	0.071	0.066	0.363	0.167	0.198
Fe % RDA 14 mg/day	0.014	0.037	0.014	0.022	0.025
Mn % RDA 10 mg/day	0.023	0.108	0.0550	0.062	0.075
Se % RDA 55 µg/day	0.001	0.002	0.001	0.001	0.001
Zn % RDA 10 mg/day	0.040	0.024	0.044	0.036	0.035
Mo % RDA 100 µg/day	0.017	0.074	0.014	0.035	0.041

## Data Availability

The original contributions presented in the study are included in the article, further inquiries can be directed to the corresponding author.

## Abbreviations

The following abbreviations are used in this manuscript:

PTWI	Provisional Tolerable Weekly Intake
PTDI	Provisional Tolerable Daily Intake
PTMI	Provisional Maximum Tolerable Daily Intake
EDI	Estimated Daily Intake
THQ	Target Hazard Quotient
HI	Hazard Index
DRVs	Dietary Reference Values
AI	Adequate Intake
AR	Average Requirement
PRI	Population Reference Intake
RI	Reference Intake Range
UL	Tolerable Upper Intake Level
MRL	Maximum Residual Levels
LOD	Limit of Detection
